# Evaluation of noise levels and noise sources in an Irish neonatal intensive care unit

**DOI:** 10.1093/annweh/wxae031

**Published:** 2024-04-23

**Authors:** Margaret McCallig, Vikram Pakrashi, Carmel Durkin

**Affiliations:** Department of Environmental Science, Atlantic Technological University, Ash Lane, Sligo F91 YW50, Ireland; School of Mechanical and Materials Engineering, Engineering Building, University College Dublin, Belfield, Dublin 5, Ireland; Neonatal Intensive Care Unit, HSE, Sligo, Ireland

**Keywords:** neonatal intensive care, noise exposure, occupational safety & health, risk assessment, training and awareness

## Abstract

**Objectives:**

This study: (i) quantified the typical noise levels in an Irish neonatal intensive care unit (NICU) and compared the values to recommendations by the American Academy of Paediatrics (AAP) and the European Standards for Care for Newborn Health (EFCNI) and to occupational exposure limit value and exposure action values; and (ii) qualified the perception of noise levels and the sources of noise across the various stakeholders within a typical NICU.

**Methods:**

A noise survey was conducted in an Irish NICU. Observations identified practices and behaviours in the NICU that potentially had an impact on noise levels. Noise levels were compared to occupational exposure limits and AAP and EFCNI standards. A noise perception survey was conducted to identify noise sources and awareness of noise levels in the NICU. Results were analysed using SPSS Statistics to determine statistical significance.

**Results:**

Noise levels recorded were consistent with previous similar studies and in all cases, the average noise levels recorded exceeded the 45 dBA as recommended by the AAP and EFCNI. There was a statistically significant difference (*P* < 0.01) between noise levels recorded on the day shift compared to the night shift. The perception of noise levels reported by nurses versus parents was found to be statistically significant (*P* = 0.001). 38.3% of all respondents reported having received no information or training with regard to noise in the NICU. There was a statistically significant difference in the perception of who is most likely to be affected by noise in the NICU, with nurses reporting those most likely to be affected by noise were patients, and parents reporting those most likely to be affected were staff (*P* = 0.003).

**Conclusions:**

This study supports the hypothesis that noise levels within the NICU are of concern and require regular assessment and monitoring. Training and awareness programmes are an important component to ensuring all persons in the NICU recognise their potential impact on noise levels in the NICU and in reducing the risk for patients and staff.

**What’s Important About This Paper?**

This is the first reported survey of noise levels in a neonatal intensive care unit in Ireland. While the measured noise levels were unlikely to negatively impact workers’ hearing, they were consistently above levels recommended for patient safety and comfort. Further education is needed for workers and patient families to understand the importance of noise reduction in this setting.

## Introduction

Noise is a very common workplace pollutant and depending on the level and duration of exposure, can cause a range of effects from annoyance, loss of concentration, and sleep disturbance to permanent noise-induced hearing loss (PNIHL) to those working in areas affected (Cherrie et al. 2021). The Safety, Health, and Welfare at Work (General Application) Regulations 2007; Chapter 1 of Part 5 set out the minimum requirements for the protection of workers from the health risks associated with workplace noise by publishing exposure action and limit values. The American Academy of Peadiatrics (AAP) recommends that NICU noise levels remain at or below 45 dBA for the most part, with maximum allowable noise levels of 65 dBA in exceptional circumstances. The European Foundation for the Care of Newborn Infants (EFCNI) publishes guidance on the management of the acoustic environment in neonatal intensive care units (NICU) which specifies components required to ensure a safe acoustic environment for neonates and staff in the NICU (Sizun et al. 2018).

The study of noise levels in neonatal intensive care units (NICUs) is a topic of concern to the paediatric community globally (Sequí-Canet et al. 2022). Noise levels in the NICU can adversely affect infants, resulting in cochlear damage, disruption of normal growth, sleep disturbances, irritability, fatigue, and general discomfort (Karlsson et al. 2012). Studies to date have determined that noise levels in NICU can have a negative effect on infants. In the hospital setting, noise is typically categorised as speech, equipment noise, therapeutic sound, and ambient noise of staff activities (Santos et al. 2015).

From a staff perspective, employers should conduct a risk assessment where exposure to noise is identified as a workplace hazard and are required to provide information to employees on the results of the risk assessment and measures taken to reduce noise exposure. For the protection of patients in the NICU, the EFCNI standard requires that parents and families are informed about the need to minimize noise to reduce discomfort for infants, and training on the same should be attended by staff working within the NICU.

## Methodology

**Fig. 1.**
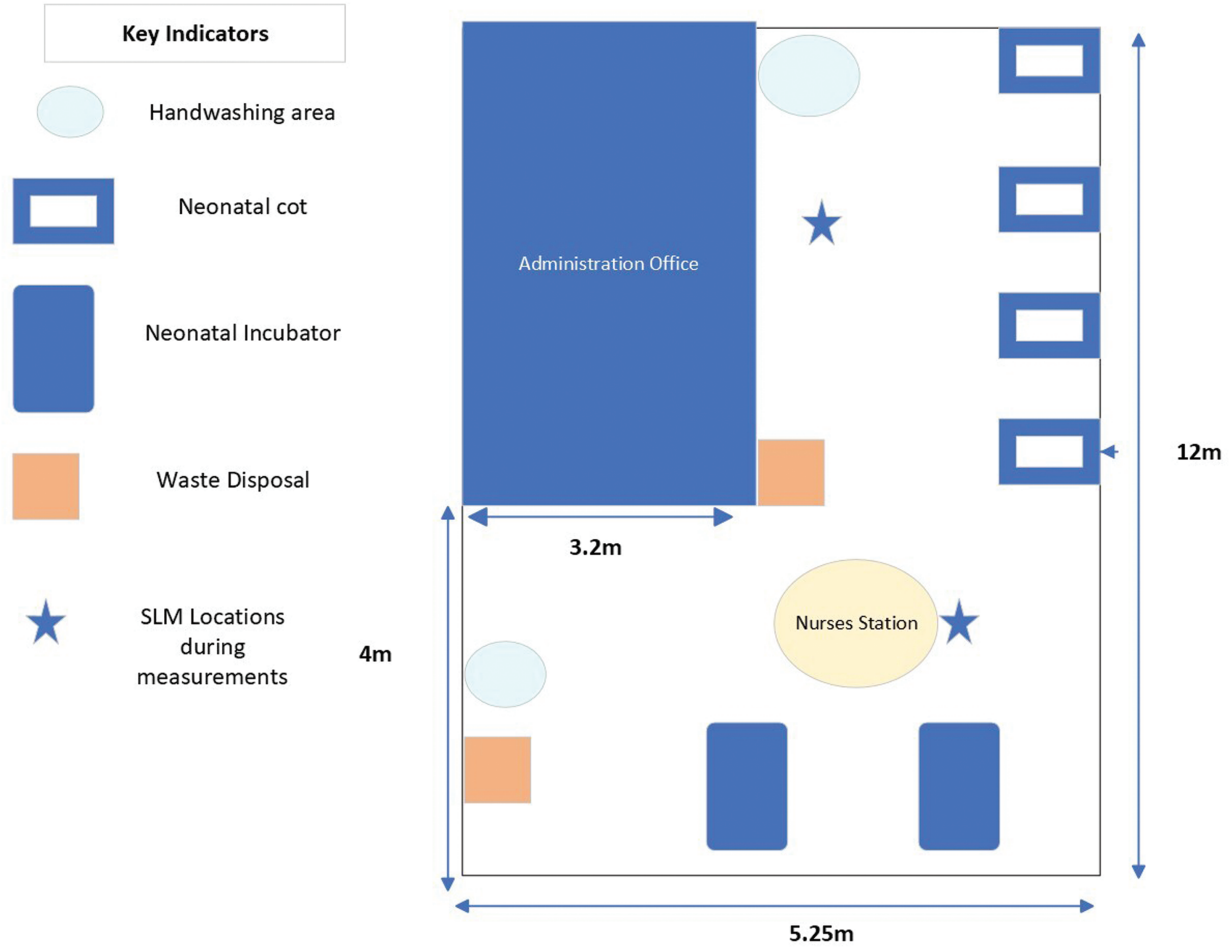
Neonatal ICU floor plan.

The research methodology for this study was approved by the Research Ethics Committee of the hospital in advance of the study commencing. This is a quantitative, descriptive and exploratory study conducted in an active NICU in Ireland. Figure 1 shows the floor plan of the unit with an active nurse’s station in the centre of the unit, and a busy administration office at the entrance to the unit.

### Noise measurement

Area noise measurements were made using Class 1 Nor131 Norsonic Sound Level Metre (SLM) over a total of 5-day shifts and 3-night shifts. The data collection times ranged from 8 h to 12 h to reflect the shift patterns of staff working in the NICU. Noise measurements were taken independently in Areas 1 and 2. The number of NICU patients, staff and parents present during measurements varied. The SLM was set up to record ‘A’ weighted slow response noise levels. The SLM was calibrated in advance in line with the manufacturer’s guidance. All equipment was sanitized before and after each use in compliance with the NICU hygiene policy. Noise levels were recorded at 2 locations in the NICU; in the center of the unit where the nurses’ station was situated (Area 1) and adjacent to the neonatal cots (Area 2) (see Fig. 1). The minimum, maximum, and average noise levels recorded by the SLM across the measurement periods were statistically analyzed using the SPSS Statistics package.

### Noise perception survey

A perception survey was completed by employees working within the NICU and the parents of patients who agreed to take part. A total of 47 perception surveys were completed, which comprised of doctors/consultants (17%), nursing staff (57.5%), professionals from other functional areas (15%), and parents (10.5%). The sample size reflects the size of the NICU where the study was undertaken. Due to the anonymity of participants, it was not possible to identify day shift staff versus night shift staff during the data analysis. The questionnaire consisted of 6 close-ended questions on the respondent’s opinion on the noise levels within the NICU, the main sources of noise and the respondent’s perception of their contribution to the noise levels. A question was included on whether the respondent had received any training or information on the noise levels within the NICU. Finally, an open-ended question was included to provide the respondents with an opportunity to outline what control measures they would put in place to reduce noise levels within the NICU. Statistical analysis on the perception survey was conducted using the SPSS Statistics package.

## Results

The results of the noise survey are presented in Table 1. In all cases, the average noise levels recorded exceeded 45 dBA as recommended by the AAP. The highest average noise level on the night shift was 54.4 dBA and the highest average noise level on the day shift was 65.7 dBA. A Shapiro–Wilk test returned a statistic *W*= 0.961, *P* = 0.676 which indicated the data was normally distributed. Further analysis using independent *t*-tests found no statistically significant difference (*P* = 0.39) between average noise levels in Area 1 versus Area 2. However, there was a statistically significant difference (*P* < 0.01) between average noise levels recorded on the day shift versus the night shift.

**Table 1.** Range (min – max) and average (dBA) noise levels recorded across day and night shifts in NICU.

Location	Shift	Range (min-max) (dBA)	Average (dBA)
Area 1	Day	40.3 to 80.1	60.2
		42.2 to 80.7	61.5
		38.8 to 77.8	58.3
		48.7 to 82.6	65.7
		41.2 to 78.9	60.1
	Night	36.4 to 68.8	52.6
		34.9 to 67.1	51.1
		38.6 to 70.1	54.4
Area 2	Day	38.5 to 72.3	55.4
		41.3 to 76.8	59.1
		36.9 to 79.1	58.2
		46.3 to 81.1	63.7
		39.6 to 77.8	58.7
	Night	35.2 to 64.7	50.1
		34.5 to 63.8	49.2
		36.3 to 68.2	52.3

Observations noted that weekly fire alarm testing and the daily emergency bleep test were attributable to the higher values recorded during the day shift versus the night shifts. Other factors noted that potentially impacted the noise levels related to human behaviour; such as loud conversation, the forced closing of waste disposal bin lids and the practice of dragging chairs across the floor rather than lifting them.

The perceived noise levels as recorded during the perception survey found 34% of respondents categorised the NICU noise levels as ‘very noisy’ or ‘noisy’. The remaining 66% of the respondents categorised the noise levels as ‘mildly noisy’ or ‘less noisy’. Mann–Whitney *U* test was used to determine if there was a statistically significant difference between the respondent categories and their perception of noise levels in the NICU. A statistically significant difference (*P* = 0.001) was recorded between the noise perception of nursing staff versus that of parents. Of all survey respondents (*N*=47), 55.3% reported that their behaviour does influence the noise levels within the NICU. More specifically, 59% of nursing staff and 63% of doctors reported that their behaviour had an impact on noise levels.

Table 2 summarises the responses to a question on whether any information with regards sources of noise and the impact of noise on staff and patients in NICU had been provided. Results show that 38.3% of respondents either ‘strongly disagreed’ or ‘disagreed’ that they had received information on noise sources and noise impact within the NICU. Similarly, 36.2% of respondents either ‘agreed’ or ‘strongly agreed’ that they had received appropriate information. Over a quarter (25.5%) of respondents neither agreed nor disagreed on whether they had received such information.

**Table 2.** Responses on whether information was provided on noise in NICU.

	Strongly disagree/disagree *N* (%)	Neutral *N* (%)	Strongly agree/agree *N* (%)	Total *N* (47)
Doctors	5(63%)	–	3(37%)	8
Nurses	9(33%)	7(26%)	11(41%)	27
Other professional	1(14%)	3(43%)	3(43%)	7
Parent/guardian	3(60%)	2(40%)	–	5

The 3 most frequently reported sources of noise within the NICU were equipment alarms (*N* = 41), staff-to-staff conversations (*N* = 37) and telephone/ call systems (*N* = 30). Respondents were asked who they perceived to most likely be negatively affected by the noise levels within the NICU. Mann–Whitney *U* test returned a statistically significant difference (*P* = 0.003) between the opinions of nurses and parents, where nursing staff indicate patients are most likely to be affected and parents indicate that staff are most likely to be affected.

Over 70% of respondents chose to answer the open-ended question on control measures to reduce noise. A summary of the responses includes a reduction in footfall within NICU, enforcement of the daily ‘quiet hour’, reduced volume levels on alarms and to erecting signage to remind people to keep noise down. The most common response was to implement an awareness campaign for staff and parents on the control and reduction of noise within the NICU.

## Discussion

The AAP and the EFCNI recommend that noise levels in the NICU should for the most part not exceed 45 dB, however average noise levels recorded in this study exceed this. The average noise levels recorded in this study as shown in Table 1 across all day and night shifts exceeded 45 dBA. Furthermore, the maximum noise levels recorded exceeded the recommended maximum of 65 dBA in all but 2 of the night shifts. Area noise levels more than the AAP recommendations have been reported across other studies (Darcy et al. 2008; Brown 2009; Degorre et al. 2017; Ahamed et al. 2018), and the excessive noise levels have been largely attributable to equipment use and staff behaviour within the NICU environment (Lasky and Williams 2009; Degorre et al. 2017). Average measured noise levels inside the NICU on a normal working day have previously been recorded as 77 ± 5 dB (Salama et al. 2011). Average noise levels ranging from 61.9 dBA to 64.8 dBA were recorded in another study across day and night shifts (Auréilo and Tochetto 2010). In this study, average noise levels on a typical day shift ranged from 55.4 to 65.7 dBA and during a typical night shift ranged from 49.2 dBA to 54.4 dBA. Noise levels recorded in this study were higher during the day shift than during the night shift; this was found to be the case elsewhere (Auréilo and Tochetto 2010). Several studies have found that noise levels frequently surpass the recommended standards and occupational legal limits for short periods of time simply due to NICU activities such as alarms sounding, chairs being dragged across floors, and opening and closing of doors (Jordao et al. 2016; Parra et al. 2017). Research suggests that staff conversations and medical equipment alarms appear to be the most disturbing noise source in an intensive care unit (Kokani et al. 2014). This study has found similarities in relation to NICU noise sources, where 2 of the most frequently reported sources of noise were equipment alarms and staff-to-staff conversations. Noise levels attributable to these activities ranging from 65 dB to 90 dB have been recorded, typically averaging at 80 dB for extended periods of time (Gray and Philbin 2000; Hoehn et al. 2000; Brandon et al. 2008). The work performed in the NICU can be particularly psychologically demanding for healthcare professionals which combined with noise exposure in the NICU can result in an increased risk of accidents and errors occurring (Carvalhais et al. 2018). Furthermore, exposure to noise may induce effects in professionals such as burnout, stress and fatigue (Mahmood et al. 2011). Evidence shows that technology combined with a high volume of healthcare professionals within the NICU creates a busy and noisy environment (Elliot et al. 2011).

A significant percentage (38.3%) of the noise perception survey respondents report having received no information or training with respect to noise, which may indicate gaps in legal compliance for hospital management as well as the potential risk to neonates. Safe noise levels are essential for effective communication and caregiving within the NICU, therefore the importance of understanding recommendations to maintain noise at a safe level should not be underestimated (Brown 2009). A further 25.5% neither agreed nor disagreed with having received such information or training. Concerningly, none of the parents surveyed indicated having received any information on noise at all despite this being a recommendation of the EFCNI standard. Previous studies suggest that neonatal nurses have a lack of expertise in noise prevention and that they would benefit from an educational program based on the reduction of noise in NICU and the impact on neonates (Aita and Goulet 2003).

A previous noise perception survey consisting of 70 participants found that 80% believed they contributed to noise on their shift (Auréilo and Tochetto 2010). Interviews with nurses to examine their perceptions of factors contributing to noise in NICU reported that most nurses perceived the NICU to be ‘pretty quiet’ (Darcy et al. 2008). Results from the noise perception survey in this study oppose the data reported in that only 23.4% of survey respondents report the NICU to be ‘less noisy’. Of the 47 respondents in this study, 55.3% reported that their behaviour within the NICU had an impact on the noise levels. This study found that according to parents, staff are most likely to be affected by noise in NICU and in contrast nurses report that patients were most likely to be affected by noise. Contrary to the findings from this survey, other research establishes that the parents of NICU patients believed that the professionals were unaffected by the noise (Auréilo and Tochetto 2010). In 1 survey which focused on mothers only, of the 95 mothers surveyed 80% considered the NICU environment as noisy. Furthermore, 50% of these mothers reported that when the NICU was noisy they had difficulty concentrating while health professionals were explaining medical interventions (Grecco et al. 2013).

The EFCNI recommends 14 components to achieve a safe acoustic NICU environment for patients and staff. Examples include reducing alarm volumes as far as is practicable, implementation of a ‘quiet hour’, a culture of minimizing noise and training on the acoustic environment for parents and for staff (Sizun et al. 2018), all of which were reported by respondents in this study when asked their opinion on how to reduce noise.

## Conclusion

Noise levels recorded in this study are not concerning from an occupational safety and health perspective for staff. However, the results do raise concerns with regard to compliance with recommendations by professional bodies and the potential impact of a noisy environment on vulnerable neonates. Regular noise monitoring should be undertaken, and the relevant control measures implemented, communicated, and monitored as part of the acoustic management within the NICU. Noise awareness and information should be provided as a priority to safeguard patients, parents, and staff in the NICU environment.

## Data Availability

The data underlying this article will be shared on reasonable request to the corresponding author.
